# Acute Effects of Particulate Matter and Black Carbon from Seasonal Fires on Peak Expiratory Flow of Schoolchildren in the Brazilian Amazon

**DOI:** 10.1371/journal.pone.0104177

**Published:** 2014-08-13

**Authors:** Ludmilla da Silva Viana Jacobson, Sandra de Souza Hacon, Hermano Albuquerque de Castro, Eliane Ignotti, Paulo Artaxo, Paulo Hilário Nascimento Saldiva, Antonio Carlos Monteiro Ponce de Leon

**Affiliations:** 1 Department of Statistics, Fluminense Federal University, Niterói, Brazil; 2 National School of Public Health, Oswaldo Cruz Foundation, Rio de Janeiro, Brazil; 3 Institute of Natural and Technological Science, Mato Grosso State University, Cáceres, Brazil; 4 Physics Institute, University of São Paulo, São Paulo, Brazil; 5 Department of Pathology, University of São Paulo, São Paulo, Brazil; 6 Department of Epidemiology, Rio de Janeiro State University, Rio de Janeiro, Brazil; The Ohio State University, United States of America

## Abstract

**Background:**

Panel studies have shown adverse effects of air pollution from biomass burning on children's health. This study estimated the effect of current levels of outdoor air pollution in the Amazonian dry season on peak expiratory flow (PEF).

**Methods:**

A panel study with 234 schoolchildren from 6 to 15 years old living in the municipality of Tangará da Serra, Brazil was conducted. PEF was measured daily in the dry season in 2008. Mixed-effects models and unified modelling repeated for every child were applied. Time trends, temperature, humidity, and subject characteristics were regarded. Inhalable particulate matter (PM_10_), fine particulate matter (PM_2.5_), and black carbon (BC) effects were evaluated based on 24-hour exposure lagged by 1 to 5 days and the averages of 2 or 3 days. Polynomial distributed lag models (PDLM) were also applied.

**Results:**

The analyses revealed reductions in PEF for PM_10_ and PM_2.5_ increases of 10 µg/m^3^ and 1 µg/m^3^ for BC. For PM_10_, the reductions varied from 0.15 (confidence interval (CI)95%: −0.29; −0.01) to 0.25 l/min (CI95%: −0.40; −0.10). For PM_2.5_, they ranged from 0.46 (CI95%: −0.86 to −0.06) to 0.54 l/min (CI95%:−0.95; −0.14). As for BC, the reduction was approximately 1.40 l/min. In relation to PDLM, adverse effects were noticed in models based on the exposure on the current day through the previous 3 days (PDLM 0–3) and on the current day through the previous 5 days (PDLM 0–5), specially for PM_10_. For all children, for PDLM 0–5 the global effect was important for PM_10_, with PEF reduction of 0.31 l/min (CI95%: −0.56; −0.05). Also, reductions in lags 3 and 4 were observed. These associations were stronger for children between 6 and 8 years old.

**Conclusion:**

Reductions in PEF were associated with air pollution, mainly for lagged exposures of 3 to 5 days and for younger children.

## Introduction

Several studies have shown adverse health effects from air pollution in many parts of the world [1]–[3]. Panel studies with children and teenagers have revealed important associations between air pollution and episodes of respiratory symptoms or lung function [4]–[8]. Such effects were found in both asthmatic and healthy subjects aged between 6 and 18 years old. Because children are still developing physiologically, exposure to air pollutants is a major risk factor for health and may have consequences in adulthood [9]. However, air pollution studies in healthy children are scarce.

In Brazil, the health effects of air pollution from biomass burning have not been thoroughly studied, especially in the Amazon region. The Brazilian Amazon dry season is the most critical period for biomass burning. From June to October, levels of particulate matter are usually above the World Health Organisation guidelines [2].

Some studies undertaken in the Amazon region have shown adverse health effects from particulate matter due to biomass burning, for instance, increases in emergency room visits, outpatient visits, and hospital admissions as well as decreases in peak expiratory flow (PEF) and increases in the frequency of micronuclei in oral epithelial cells [10]–[15]. These effects were found in children and the elderly in municipalities around the Arc of Deforestation region.

The Brazilian Amazon covers an area of approximately 5,000,000 km^2^ and 9 Brazilian states. Over 25 million inhabitants are changing the land use [16]. The majority of forest fire hotspots takes place in the Arc of Deforestation [17], whilst the state of Mato Grosso usually shows the worst records. Ignotti et al. [17] compared the indicators of morbidity and mortality caused by respiratory diseases in municipalities of Mato Grosso. Alta Floresta and Tangará da Serra were identified as priority areas for assessing the air pollution health effects of biomass burning in the Brazilian Amazon.

A panel study of schoolchildren carried out in the municipality of Alta Floresta found adverse effects of particulate matter with an aerodynamic diameter less than 2.5 µm (PM_2.5_) on PEF [10]. The results were stratified by the time of day the children attended school, morning or afternoon. Stronger associations were detected in the afternoon group. However, only single-lag exposures were estimated, namely, levels of PM_2.5_ on the current day or lagged by 1 or 2 days and the averages of 0- to 1-day lags, 1- to 2-day lags, and 0- to 2-day lags.

A similar panel study was carried out in Tangará da Serra in 2008. Its results are reported in this article. The primary aim of this study was to estimate the effect of current air pollution levels in the Amazonian dry season on schoolchildren's PEF. Three separate secondary aims were investigated, namely:

- To compare two methodological approaches in the analysis of repeated measures data: mixed-effects models (MEM) and a unified model to be applied for every child;
- To assess the role played by age in explaining the difference between the morning and afternoon groups of children that Jacobson et al. [10] uncovered. In Brazil, most primary schools adopt the policy of two periods of study classes, morning and afternoon. Further, younger children attend school in the afternoon and older children go to school in the morning. Therefore, age could explain the difference observed in the previous study;
- To extend the lag structures that Jacobson et al. [10] explored but to also use a polynomial distributed-lag model (PDLM). In the Amazon, particles emitted by biomass burning remain in the atmosphere for one week or so [18]. Therefore, to evaluate the cumulative effects based on polynomial distributed-lag models seems to be more appropriate. Moreover, few panel studies have used PDLM to estimate air pollution effects, especially those related to children's PEF [19]–[21].

## Material and Methods

### Study Design and Variables

**Figure 1:**
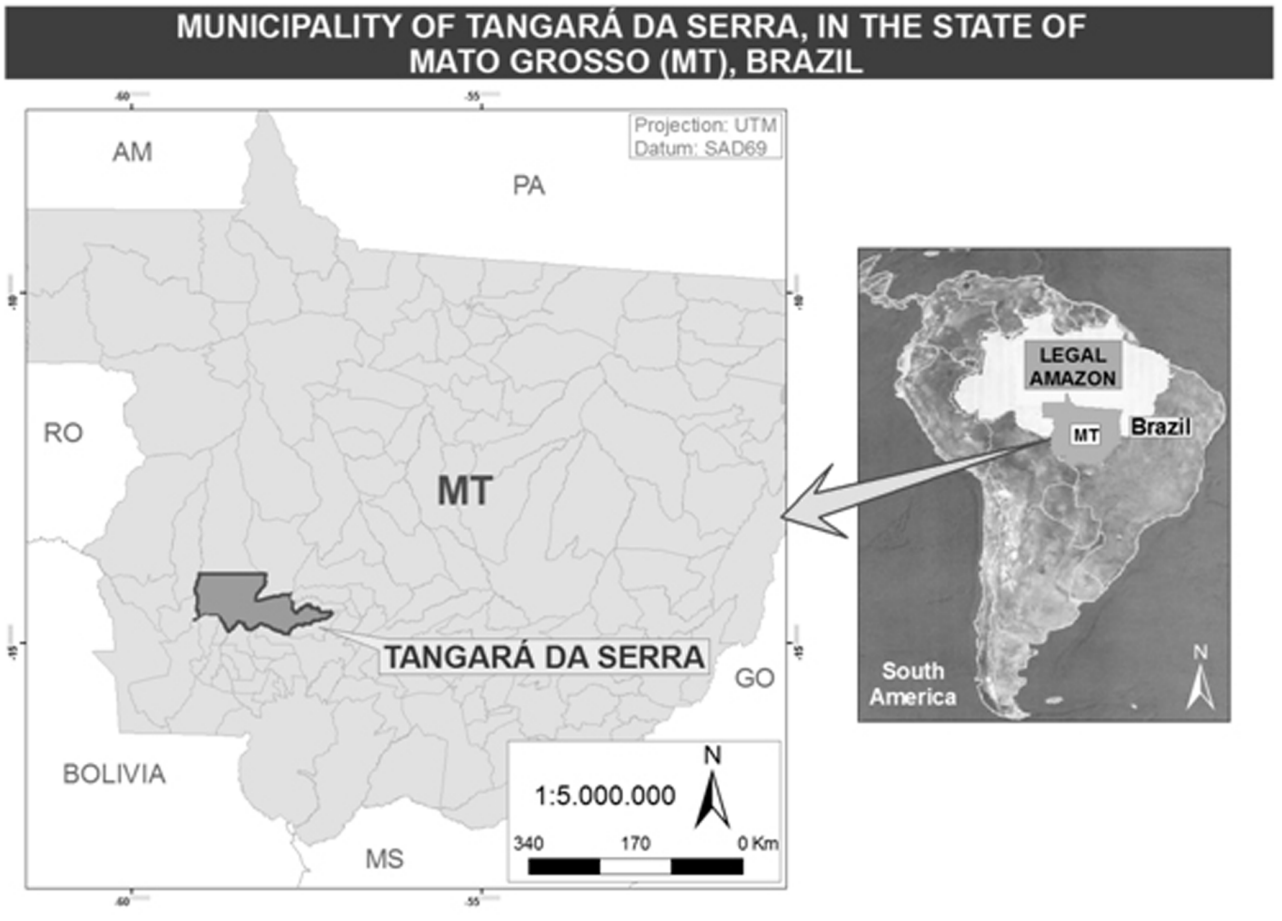
Geographic location of the municipality of Tangará da Serra, Mato Grosso State.

The study was conducted in the Tangará da Serra municipality, in the southwest of Mato Grosso State, in a transition area of the Amazon and Cerrado biomes (Figure 1). Its total population was 83,431 inhabitants (population density was 7.32 inhab./km^2^), and the population from 6 to 15 years old was 14,398 inhabitants (17.3%) [16]. Its main economic activities are agriculture and livestock. Despite its increasing production of sugarcane, the harvest is still manual and occurs during the dry season [22]. The pre-harvest burning of sugarcane and the plumes of biomass burnings from neighbouring regions are the main sources of air pollution in the city.

In 2008, a panel study of schoolchildren from a local public school was carried out (Lat 14°37′59.05′′ S; Long 57°31′25.34′′ W). The school had 875 students from 6 to 15 years old attending school in the morning or in the afternoon. The study sample comprised 234 schoolchildren who were followed up daily, except weekends and holidays, from August to November. Children were randomly selected according by age group (between 6 and 15) and school schedule.

Ethical approval was obtained from the UNEMAT (Mato Grosso State University) Ethics Committee, and a consent form to participate in the study was signed by the children's parents or legal guardians. The protocol described by the American Thoracic Society [23] for PEF exams was adopted throughout the study. After careful training, nursing undergraduate students collected PEF data under the supervision of a senior pulmonologist and a senior nurse.

Three daily PEF measures were sequentially taken. To avoid disruption of the school routine, groups of 4 or 5 children performed the tests and returned to class afterwards. The device used was the Mini-Wright Peak Flow Meter (Clement Clarke International Ltd, United Kingdom). The study outcome was the maximum of the three sequential PEF measures.

Weight and height were measured at the study's onset, using a Welmy anthropometric mechanical scale with 150 kg maximum capacity. Face-to-face questionnaires comprising health conditions and exposure to tobacco smoking at home were answered by the children's guardians/parents. To assess asthma symptoms, the questionnaire included phase I of the *International Study of Asthma and Allergies in Childhood* [24].

Exposure to air pollution was measured during the entire study period. The devices were placed at the UNEMAT campus in Tangará da Serra. The selected pupils lived within a 5 km radius of the campus and a maximum of 1 km radius of the study school. PM_10_ concentrations (particulate matter with an aerodynamic diameter less than 10 µm) were measured continuously using the Thermo Electron Corporation (DATARAM) device. Moreover, the Aethalometer was used to measure black carbon (BC) levels, and stacked-filter units (SFU) were also used to measure PM_2.5_. The following were regarded as exposures to PM_10_ and BC: (i) the 24-hour mean of the previous day (lag 1) until 5 days before (lag 5); and cumulative exposures, such as the average of the daily means of the same day and the previous one (lags 0–1), of the previous two days (lags 1–2), and of the same day and the two previous days (lags 0–2). For PM_2.5_, there were a few missing data; therefore, to minimise loss of information, cumulative exposures were not investigated.

Measures of relative humidity and temperature were provided by INMET (National Institute of Meteorology, Brazil). Levels of relative humidity and mean temperature were regarded in all statistical models.

### Statistical Analysis

The study database included variables that vary over time, for example, the outcome, the exposure, the weather, and outdoor leisure time, but also time-invariant covariates, such as age, sex, passive smoking, asthma, weight, height, and body mass index (BMI). Despite the data collection from August to November, this study presents the results for the dry season period only, from August to October.

Five types of statistical analysis were implemented in order of complexity, namely: (i) separate data analysis for each child; (ii) a mixed-effects core model regarding climate and individual covariates; (iii) air pollution effect estimation for single lags; (iv) air pollution effect estimation for PDLM; (v) and a sensitivity analysis.

For item (i), the same modelling steps were applied to the PEF time series for each child. Several strategies were examined for the adjustment of time trends, temperature and humidity, such as linear, quadratic or parametric splines with one knot or two knots, as well as linear or quadratic polynomials. The modelling proceeded including a 1^st^ order autoregressive term, later estimated for each child. To summarise the results, averages were calculated for the Akaike Information Criterion (AIC), the estimate of the 1^st^ order autoregressive term (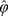), and the set of PM_10_ lag 1 effects. Positive (p<0.05), negative (p<0.05), or null effects (p>0.05) of PM_10_ lag 1 were classified into three groups. The unified model that presented the smallest AIC average was selected. Finally, tables of counts (proportions) or averages by sex, age, passive smoking, presence of asthma, BMI, weight, and height allowed comparison of the three groups.

Because this was a study of repeated measures, MEMs were also appropriate for the statistical analysis. Regarding this approach, the model adjustment considered a common autoregressive lag 1 (AR(1)) correlation structure whereas the variance function comprised three time-invariant covariates, namely, BMI in four categories, age in three, and asthma status. Four adjustments based on polynomials of time were evaluated for the outcome long-term trend: a cubic parametric spline with one knot; a quadratic parametric spline with two equally spaced knots; a quadratic parametric spline with one knot; and a quadratic polynomial of time [25]. The above considered time to be centred in the middle of the study period as well as random coefficients. The same approach was applied for temperature and relative humidity, but also linear effects were explored. Furthermore, exposures on the same day, the previous day, and the day before that were examined. Finally, height, weight, BMI, age, sex, ISAAC asthma status, passive smoking, and outdoor leisure time, with fixed effects, were regarded in the models. Because of the number of model comparisons, the AIC and the deviance test were used to select the most parsimonious model, which then was scrutinised with regard to residual diagnostics to confirm the normality assumptions of the random effects. Jacobson et al.[10] describe the details of this approach.

Regarding the choice of core model based on the modelling steps mentioned above, single-lag and PDLM were applied to estimate the effects. Single-lag models were fitted for all exposures mentioned previously. PDLM estimates a fixed number of lagged effects constrained to a given choice of polynomial as well as the overall sum of effects [26]. In this study, quadratic polynomials of the effects lagged up to 3 (0–3) or 5 (0–5) days were calculated.

Sensitivity analyses were performed to assess the robustness of the results. For instance, measures of PM_2.5_ were missing for 25% of the study days. Therefore, missing values of PM_2.5_ were replaced by the PM_2.5_ average on the previous day and the day after. The effect of this exposure on PEF was estimated using single lags and PDLM. Because of the methods used in PDLM, we evaluated the effects of PM_2.5_ only considering levels after imputation. Furthermore, all of the analyses were repeated excluding outliers, the definition of which was standardised residuals greater than four in absolute value. We also explored the presence of a threshold effect of PM_10_ using a linear parametric spline. Each exposure (lag 1, lag 2, lag 3, lag 4, lag 5, and cumulative lag 1–5) was centred at different thresholds, namely, 20 µg/m^3^, 30 µg/m^3^, 40 µg/m^3^, 50 µg/m^3^, 60 µg/m^3^, 70 µg/m^3^, and 80 µg/m^3^.

All analyses were performed using R 2.15.1 [27] and the library *nlme* [28].

## Results

Of the 234 students selected for the sample, 7 left school during the study and were considered lost to follow-up. A group of 7 students were excluded from the regression analysis because their guardians/parents did not answer the questionnaire. The total number of study days was 53.

Table 1 displays the summary statistics of individual characteristics (sex, age, weight, height, asthma, and passive smoking), PEF measures, and weather and air pollution variables. Forty-seven percent of the 227 students were boys, the average age was 10.3 years old, the average weight was 36.2 kg (17 to 82.2 kg), and the average height was 1.40 m (1.11 to 1.73 m). According to the ISAAC criteria, 18% of the students (n = 39) reported asthma symptoms (21 girls and 18 boys). Passive smoking at home was reported by 33% of the students' parents (n = 72). Overall mean PEF was 289.9 l/m and it ranged from 70 to 780 l/min.

**Table 1:** Summary statistics of weather variables, PM_10_, PM_2.5_, BC, peak expiratory flow measurements, and children's individual characteristics.

Variables					Percentile					
	n	%missing	average (sd)	min	10	25	50	75	90	max
Average daily temperature (°C)	82	0.0	26.0 (2.8)	16.5	22.2	24.2	26.8	28.0	29.1	29.9
Average daily humidity (%)	82	0.0	56.8 (17.1)	29.4	34.2	42.0	54.9	70.0	81.6	90.5
Average daily PM_10_ (µg/m^3^)	75	8.5	62.7 (40.7)	12.4	20.5	29.7	52.7	89.7	127.3	164.5
Average daily PM_2.5_ (µg/m^3^)	61	25.6	19.6 (11.9)	4.4	6.6	9.6	16.1	28.3	35.8	55.9
Average daily black carbon (µg/m^3^)	72	12.2	0.998 (0.481)	0.271	0.403	0.597	0.949	1.254	1.834	2.146
Peak flow (l/m)	11198	41.14*	289.9	70.0	180.0	220.0	280.0	350.0	410.0	780.0
Age	227	0.0	10.3 (2.5)	6.0	7	8	10.0	13.0	14.0	15.0
Weight (Kg)	227	0.0	36.2 (21.4)	17.0	21.4	26.1	32.8	43.5	55.4	82.2
Height (cm)	227	0.0	140.2 (120.0)	111.0	120.0	129.0	138.0	152.0	162.0	173.0
BMI	227	0.0	17.9 (3.5)	11.9	14.5	15.2	16.9	19.7	22.7	32.1
Asthmatic (%)	220	3.08	18.0							
Passive smoke (%)	218	4.0	33.0							
Boys (%)	227	0.0	46.7							

Tangará da Serra-MT, Brazil, 2008.

n = number of observations; sd = standard deviation; min = minimum; max = maximum; * includes holidays, weekends and actual missing values.

**Figure 2:**
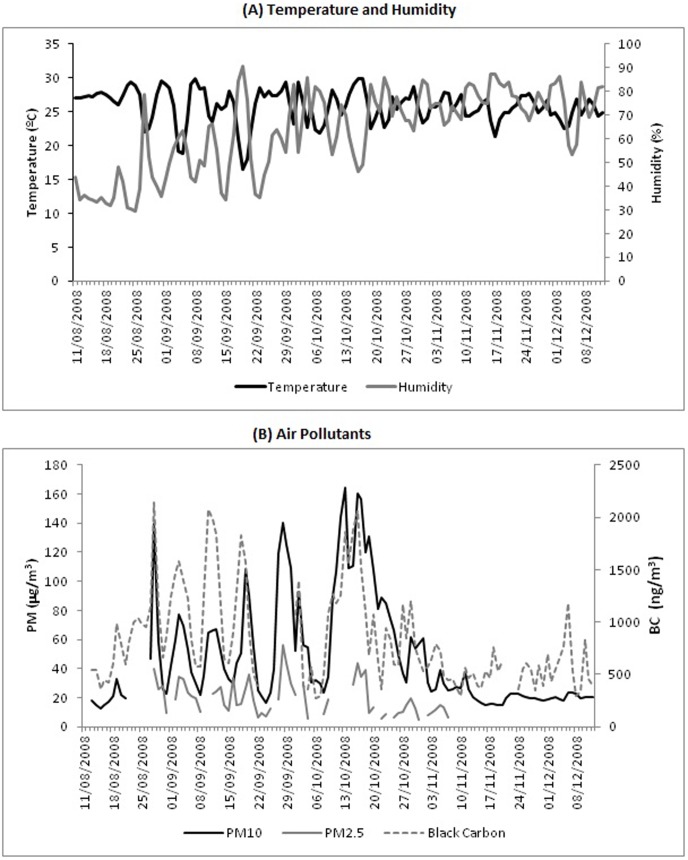
Daily temperature, humidity and air pollutant readings. Tangará da Serra, MT, Brazil-2008.

The average relative humidity and the daily mean temperature were 57% (29.4 to 90.5%) and 26°C (16.5 to 29.9°C), respectively. The averages of the daily mean PM_10_, PM_2.5_, and BC were 62.7 µg/m^3^ (12.4 to 164.5 µg/m^3^), 19.6 µg/m^3^ (4.4 to 55.9 µg/m^3^), 0.998 µg/m^3^ (0.271 to 2.146 µg/m^3^), respectively (Table 1). Typically in this part of Brazil in October and November, humidity levels increase and air pollution levels decrease because of the beginning of the rainy season, whereas temperature levels are somewhat constant. Figure 2 shows the time series for temperature, humidity, PM_10_, PM_2.5_, and BC.

In this section, the effects reported are in litres per minute and are for increases of 10 µg/m^3^ for PM_10_ and PM_2.5_, and 1 µg/m^3^ for BC.

### Results for the Unified Model Approach for Each Child

Table 2 shows the summary statistics of the unified model approach. Regarding AIC, 1^st^ order autocorrelation (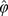), PM_10_ lag 1 effect averages, and counts of individuals with positive or negative effects of PM_10_ lag 1 are displayed. The most parsimonious 1^st^ order autoregressive model consisted of a quadratic parametric spline with two equally spaced knots for the long-term trend, a quadratic polynomial for humidity lagged by two days, and temperature lagged by two days. The 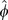 average was 0.0232 (sd 0.246; −0.812 to 0.549), and for 34 children the fitted models presented 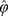 larger than zero and p<0.05. With respect to PM_10_ lag 1, 12 students presented positive effects (p<0.05), 13 students presented adverse effects (p<0.05), and 202 presented no effects (p≥0.05).

**Table 2:** Summary adjustment strategies of the unified model approach.

Model Adjustment – AR(1) (n = 227)			AIC	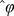		Change in PEF (PM10 lag 1)***		
Time trend	Humidity (lag 2)	Temperature (lag 2)	Average*	Average*	p<0.05 and 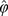 higher than zero (n)	Average **	Positive and p<0.05 (n)	Negative and p<0.05 (n)
Linear Spline	Linear	Linear	401.74	0.1468	61	0.038	11	10
Linear Spline	Linear Spline	Linear Spline	402.91	0.1453	57	0.047	15	12
Quadratic Polynomial	Quadratic Polynomial	Quadratic Polynomial	403.17	0.1483	65	0.027	15	12
Quadratic Spline^⊥^ (1 Knot)	Quadratic Polynomial	Quadratic Polynomial	402.49	0.0854	46	0.039	17	13
Quadratic Spline^⊥^ (2 Knot)	Quadratic Polynomial	Quadratic Polynomial	401.41	0.0173	35	−0.017	15	12
*Quadratic Spline^⊥^ (2 Knot)*	*Quadratic Polynomial*	*Linear*	*400.83*	*0.0232*	*34*	*−0.033*	*12*	*13*
Cubic Spline^⊥^ (1 Knot)	Quadratic Polynomial	Quadratic Polynomial	401.78	0.0270	35	−0.035	14	13

Tangará da Serra-MT, Brazil, 2008.

*Simple arithmetic average; **Averages weighed by the inverse of their variances; *** Change in PEF for an increase of 10 µg/m^3^ in PM_10_lag1; n – Number of cases; ^⊥^Parametric Splines.

Table 3 summarizes the results of the AR(1) estimated model according to individuals characteristics. Averages of weight, height, BMI, and age were calculated according to these groups (positive, negative, and non-significant effect) and compared using the Kruskal-Wallis test. Of the four individual covariates, only age showed differences (p<0.05). Similarly, proportions of sex, asthma, and passive smoking were compared across groups using Fisher's exact test. There were no proportion differences. Of the individual covariates investigated, only age affected the magnitude of PM_10_ effects (Table 3).

**Table 3:** Characteristics of the schoolchildren according to the results of the AR(1) estimated model.

Individual Characteristics	Change in PEF - PM_10_ Lag1		
	Positive and Significant (n = 12)	Negative and Significant (n = 13)	Non-Significant (n = 202)
Sex (%)			
Girls	66.7 (n = 8)	46.2 (n = 6)	53.0 (n = 107)
Boys	33.3 (n = 4)	53.8 (n = 7)	47.0 (n = 95)
Asthma Status (%)			
Yes	8.3 (n = 1)	23.1 (n = 3)	18.1 (n = 35)
No	91.7 (n = 11)	76.9 (n = 10)	81.9 (n = 158)
Passive Smoke (%)			
Yes	33.3 (n = 4)	30.8 (n = 4)	33.2 (n = 64)
No	66.7 (n = 8)	69.2 (n = 9)	66.8 (n = 129)
Age			
Average (SD)	12 (2.17)	9.62 (2.43)	10.23 (2.55)
Weight			
Average (SD)	43.57 (15.01)	35.26 (11.42)	35.92 (13.40)
Height			
Average (SD)	149.3 (14.16)	138.69 (11.93)	139.71 (15.28)
BMI			
Average (SD)	19.05 (3.87)	17.97 (4.03)	17.79 (3.50)

Tangará da Serra-MT, Brazil, 2008.

Moreover, we computed combined-effect estimates of the exposure to PM_10_ levels lagged by between 1 and 5 days, using averages weighed by the inverse of their variances. The most adverse effect found was for lag 4. For PM_10_ lag 4, there was a reduction in PEF equal to 0.16 (95% confidence interval(CI): −0.31; −0.02). The other results are not shown.

### Results for the Mixed-Effects Models

MEMs were applied in the next step of the data analysis. The choice of core model consisted of long-term trend adjusted by a quadratic parametric spline with two equally spaced knots and random coefficients, a quadratic polynomial curve for humidity lagged by two days with random coefficients, temperature lagged by two days and a random coefficient, and fixed-effects covariates such as BMI, age, sex, asthma status, and exposure to air pollution; it also regarded an AR(1) and sixteen different subject-error variances depending on the subject status with respect to asthma, age, and BMI. Table 4 summarises the adjusted mixed models.

**Table 4:** Summary adjustment strategies of the mixed-effect models.

Model Adjustment* (n = 220)			AIC	PHI (AR(1))	Change in PEF **(IC95%)
Time trend	Humidity (lag 2)	Temperature (lag 2)			
Linear	Linear	Linear	90301.19	0.332	−0.020 (−0.165; 0.125)
Linear	Quadratic Polynomial	Linear	90292.05	0.330	−0.021 (−0.165; 0.123)
Linear Spline⊥	Linear	Linear	90207.98	0.291	−0.016 (−0.157; 0.125)
Quadratic Polynomial	Quadratic Polynomial	Linear	90217.62	0.295	−0.026 (−0.168; 0.115)
Quadratic Spline⊥ (1 Knot)	Quadratic Polynomial	Linear	90147.36	0.261	−0.048 (−0.188; 0.092)
*Quadratic Spline* ⊥ *(2 Knot)*	*Quadratic Polynomial*	*Linear*	*90104.27*	*0.238*	*−0.108 (−0.251; 0.035)*
Cubic Spline⊥ (1 Knot)	Quadratic Polynomial	Linear	90120.02	0.242	−0.114 (−0.259; 0.032)

Tangará da Serra-MT, Brazil, 2008.

*Model adjusted for age, BMI, gender, and asthma status; random coefficients for the intercept, time trend, humidity, and temperature; variance function of the random error included age, BMI, and asthma status.

** Change in PEF for an increase of 10 µg/m^3^ in PM_10_lag1.

⊥ Parametric Splines.

### Single-Lag Effects

Table 5 displays the results of the models for all students and stratified by three age groups and considering single-lag exposures. This approach revealed some adverse effects of air pollution on PEF. Taking into account all children: (i) for PM_10_, the results showed reductions of 0.25 (95%CI: −0.40; −0.10), 0.20 (95%CI: −0.32; −0.07), and 0.15 (95%CI: −0.29; −0.01) in lags 3, 4, and 5, respectively; (ii) for PM_2.5_ lag 4, there was a reduction of 0.54 (95%CI: −0.95; −0.14). However, after PM_2.5_ imputation, the models revealed associations not only with lag 4 (−0.50; 95%CI: −0.89; −0.12) but also with lag 3 (−0.46; 95%CI: −0.86; −0.06); (iii) for BC, the reduction was roughly 1.40 (95%CI: −2.50; −0.29) in both lags 4 and 5.

**Table 5:** Estimated changes in peak expiratory flow (in l/min) for an increase of 10 µg/m^3^ in PM_10_ and PM_2.5_ and an increase of 1 µg/m^3^ in black carbon for all children and stratified by age groups.

Exposure	All Children (n = 220)		6 to 8 years (n = 69)		9 to 11 years (n = 69)		12 to 15 years (n = 82)	
	Change in PEF (95%CI)		Change in PEF (95%CI)		Change in PEF (95%CI)		Change in PEF (95%CI)	
**PM_10_**								
Lag 1	−0.108	(−0.251; 0.035)	−0.215	(−0.436; 0.006)	−0.102	(−0.375; 0.172)	0.082	(−0.175; 0.338)
Lag 2	−0.104	(−0.251; 0.042)	−0.280	(−0.504; −0.055)	−0.015	(−0.301; 0.271)	0.100	(−0.162; 0.362)
Lag 3	−0.252	(−0.399; −0.104)	−0.427	(−0.654; −0.199)	−0.086	(−0.376; 0.205)	−0.112	(−0.372; 0.148)
Lag 4	−0.196	(−0.322; −0.070)	−0.283	(−0.477; −0.089)	−0.139	(−0.385; 0.108)	−0.125	(−0.347; 0.097)
Lag 5	−0.151	(−0.293; −0.010)	−0.296	(−0.515; −0.077)	−0.115	(−0.393; 0.162)	−0.055	(−0.304; 0.195)
Lag 0–1	−0.071	(−0.245; 0.103)	−0.171	(−0.441; 0.099)	−0.067	(−0.40; 0.266)	0.117	(−0.191; 0.426)
Lag 1–2	−0.126	(−0.284; 0.032)	−0.298	(−0.540; −0.055)	−0.063	(−0.370; 0.245)	0.107	(−0.174; 0.388)
Lag 0–2	−0.104	(−0.287; 0.078)	−0.262	(−0.545; 0.021)	−0.049	(−0.406; 0.309)	0.123	(−0.201; 0.447)
**PM_2.5_**								
Lag 1	0.129	(−0.316; 0.573)	0.108	(−0.576; 0.791)	0.084	(−0.748; 0.915)	0.175	(−0.590; 0.941)
Lag 2	−0.286	(−0.773; 0.201)	−0.905	(−1.646; −0.163)	0.202	(−0.700; 1.103)	0.132	(−0.702; 0.966)
Lag 3	−0.377	(−0.792; 0.037)	−0.322	(−0.954; 0.311)	−0.558	(−1.331; 0.216)	−0.191	(−0.926; 0.544)
Lag 4	−0.541	(−0.946; −0.137)	−0.931	(−1.545; −0.317)	−0.560	(−1.296; 0.175)	−0.159	(−0.866; 0.549)
Lag 5	−0.029	(−0.487; 0.429)	−0.137	(−0.835; 0.560)	0.297	(−0.556; 1.150)	−0.403	(−1.212; 0.406)
**PM_2.5_ Imputation**								
Lag 1	0.134	(−0.283; 0.550)	0.042	(−0.603; 0.688)	0.269	(−0.531; 1.070)	0.176	(−0.566; 0.918)
Lag 2	−0.181	(−0.601; 0.239)	−0.581	(−1.227; 0.065)	0.443	(−0.366; 1.252)	−0.016	(−0.770; 0.738)
Lag 3	−0.462	(−0.862; −0.062)	−0.433	(−1.043; 0.178)	−0.515	(−1.294; 0.263)	−0.253	(−0.965; 0.459)
Lag 4	−0.504	(−0.889; −0.118)	−0.874	(−1.466; −0.283)	−0.575	(−1.326; 0.175)	−0.087	(−0.775; 0.600)
Lag 5	−0.051	(−0.478; 0.377)	−0.474	(−1.135; 0.186)	0.201	(−0.620; 1.022)	−0.017	(−0.776; 0.743)
Lag 0–1	0.261	(−0.231; 0.753)	0.298	(−0.463; 1.058)	0.301	(−0.644; 1.246)	0.242	(−0.636; 1.122)
Lag 1–2	−0.094	(−0.616; 0.429)	−0.480	(−1.127; 0.749)	0.509	(−0.496; 1.514)	0.050	(−0.876; 0.975)
Lag 0–2	0.048	(−0.556; 0.651)	−0.189	(−1.127; 0.749)	0.535	(−0.623; 1.693)	0.115	(−0.952; 1.181)
**BC**								
Lag 1	0.610	(−0.435; 1.655)	1.950	(0.323; 3.577)	0.120	(−1.762; 2.002)	−0.730	(−2.592; 1.132)
Lag 2	0.074	(−1.284; 1.432)	0.630	(−1.467; 2.727)	0.470	(−1.980; 2.920)	−1.330	(−3.760; 1.100)
Lag 3	−0.277	(−1.635; 1.081)	−0.900	(−2.978; 1.178)	0.640	(−1.810; 3.090)	−0.430	(−2.880; 2.020)
Lag 4	−1.396	(−2.499; −0.293)	−2.140	(−3.826; −0.454)	−1.200	(−3.180; 0.780)	−0.060	(−2.059; 1.939)
Lag 5	−1.539	(−2.525; −0.553)	−2.510	(−4.019; −1.001)	−1.880	(−3.644; −0.116)	0.200	(−1.584; 1.984)
Lag 0–1	1.428	(0.230; 2.626)	2.630	(0.768; 4.492)	0.940	(−1.216; 3.096)	0.080	(−2.037; 2.197)
Lag 1–2	0.620	(−0.705; 1.945)	1.920	(−0.158; 3.998)	0.370	(−2.002; 2.742)	−1.070	(−3.442; 1.302)
Lag 0–2	1.410	(−0.005; 2.825)	2.854	(0.639; 5.069)	0.950	(−1.578; 3.478)	−0.310	(−2.819; 2.199)

Tangará da Serra-MT, Brazil – 2008.

The results were stratified by age: 6 to 8 years old, 9 to 11, and 12 to 15. Children aged 6–8 were the most susceptible group. The results regarding this group were: (i) for PM_10_, the reduction varied from 0.28 (95%CI: −0.50; −0.06) to 0.43 (95%CI: −0.65; −0.20) in lags 2, 3, 4, and 5, and there was also a reduction of 0.30 (95%CI: −0.54; −0.06) for the cumulative exposure in lag 1–2; (ii) for PM_2.5_, important reductions were observed for lag 2 (−0.91; 95%CI: −1.65; −0.16) and lag 4 (−0.93; 95%CI: −1.55; −0.32). After imputation, the adverse effect of PM_2.5_ was noticed only for lag 4 (−0.87; 95%CI: −1.47; −0.28); (iii) for BC, lagged exposures of 4 and 5 days reduced PEF in this age group. Further, for children aged 9–11, the adverse effect was observed only for BC. For lag 5, there was a reduction of 1.88 (95%CI: −3.64; −0.12). No associations were found for children aged 12–15.

### Polynomial Distributed-lag Effects

Table 6 presents the results of the PDLM 0–3 for all children and stratified by age. Some adverse effects were revealed under this approach. For PM_10_, the overall effect was not significant for all children; however, lag 3 showed a reduction of 0.25 (95%CI: −0.43; −0.08). There was also an overall adverse effect of PM_10_ for children 6–8 (−0.34; 95%CI: −0.66; −0.01), particularly for lag 3 (−0.35; 95%CI: −0.62; −0.08). For PM_2.5_ lag 3, the group of all children suffered a reduction of 0.41 (95%CI: −0.81; −0.01). There were no adverse effects for BC. Likewise, no evidence of adverse effects was found for children aged 9–15.

**Table 6:** Estimated changes in peak expiratory flow (in l/min) for an increase of 10 µg/m^3^ in PM_10_ and PM_2.5_ and an increase of 1 µg/m^3^ in black carbon for all children and stratified by age groups, according to PDLM based on the exposures of the current day to the previous 3 days.

Exposure	All Children (n = 220)		6 to 8 years (n = 69)		9 to 11 years (n = 69)		12 to 15 years (n = 82)	
	Change in PEF (95%CI)		Change in PEF (95%CI)		Change in PEF (95%CI)		Change in PEF (95%CI)	
**PM_10_**								
Lag 0	0.037	(−0.161; 0.236)	0.125	(−0.174; 0.425)	0.047	(−0.321; 0.414)	−0.107	(−0.465; 0.252)
Lag 1	0.066	(−0.054; 0.186)	0.023	(−0.161; 0.207)	−0.034	(−0.258; 0.190)	0.206	(−0.006; 0.419)
Lag 2	−0.030	(−0.150; 0.089)	−0.135	(−0.316; 0.046)	−0.066	(−0.288; 0.156)	0.148	(−0.068; 0.364)
Lag 3	−0.252	(−0.429; −0.075)	−0.349	(−0.623; −0.076)	−0.050	(−0.381; 0.281)	−0.281	(−0.591; 0.028)
Overall	−0.179	(−0.390; 0.031)	−0.336	(−0.661; −0.010)	−0.104	(−0.496; 0.288)	−0.034	(−0.402; 0.335)
**PM_2.5_**								
Lag 0	0.199	(−0.270; 0.669)	0.561	(−0.147; 1.269)	−0.072	(−0.926; 0.781)	0.018	(−0.834; 0.870)
Lag 1	0.084	(−0.210; 0.378)	−0.163	(−0.613; 0.288)	0.368	(−0.170; 0.906)	0.121	(−0.402; 0.645)
Lag 2	−0.119	(−0.432; 0.195)	−0.443	(−0.921; 0.034)	0.216	(−0.357; 0.789)	0.012	(−0.549; 0.574)
Lag 3	−0.408	(−0.809; −0.007)	−0.281	(−0.893; 0.331)	−0.528	(−1.266; 0.209)	−0.308	(−1.022; 0.406)
Overall	−0.243	(−0.922; 0.435)	−0.326	(−1.373; 0.720)	−0.016	(−1.267; 1.234)	−0.156	(−1.352; 1.040)
**BC**								
Lag 0	1.797	(0.504; 3.090)	1.760	(−0.226; 3.746)	1.774	(−0.511; 4.059)	1.753	(−0.574; 4.082)
Lag 1	0.111	(−0.719; 0.942)	0.963	(−0.309; 2.235)	−0.286	(−1.751; 1.179)	−0.771	(−2.278; 0.737)
Lag 2	−0.566	(−1.484; 0.352)	0.069	(−1.355; 1.494)	−0.733	(−2.353; 0.888)	−1.304	(−2.945; 0.336)
Lag 3	−0.235	(−1.649; 1.179)	−0.920	(−3.072; 1.232)	0.434	(−2.066; 2.935)	0.152	(−2.433; 2.737)
Overall	1.108	(−0.739; 2.955)	1.873	(−1.031; 4.777)	1.189	(−2.091; 4.470)	−0.169	(−3.427; 3.089)

Tangará da Serra-MT, Brazil – 2008.

The PDLM 0–5 effect estimates revealed stronger evidence of adverse effects (Table 7). Regarding PM_10_ for all children, the overall effect was −0.31 (95%CI: −0.56; −0.05). Additionally, reductions of 0.09 (95%CI: −0.16; −0.01) and 0.10 (95%CI: −0.15; −0.05) in lags 3 and 4, respectively, were estimated. For children aged 6–8, there were strong effects for lags 2, 3, and 4. The overall effect for this age group indicated a reduction of 0.52 (95%CI: −0.92; −0.12) caused by PM_10_.

**Table 7:** Estimated changes in peak expiratory flow (in l/min) for an increase of 10 µg/m^3^ in PM_10_ and PM_2.5_ and an increase of 1 µg/m^3^ in black carbon for all children and stratified by age groups, according to PDLM based on the exposures of the current day to the previous 5 days.

Exposure	All Children (n = 220)		6 to 8 years (n = 69)		9 to 11 years (n = 69)		12 to 15 years (n = 82)	
	Change in PEF (95%CI)		Change in PEF (95%CI)		Change in PEF (95%CI)		Change in PEF (95%CI)	
**PM_10_**								
Lag 0	0.046	(−0.111; 0.202)	0.076	(−0.162; 0.313)	−0.039	(−0.330; 0.252)	0.040	(−0.241; 0.321)
Lag 1	0.012	(−0.046; 0.070)	−0.045	(−0.134; 0.045)	−0.022	(−0.130; 0.087)	0.038	(−0.064; 0.140)
Lag 2	−0.056	(−0.125; 0.013)	−0.124	(−0.229; −0.019)	−0.020	(−0.149; 0.108)	0.022	(−0.102; 0.145)
Lag 3	−0.085	(−0.158; −0.012)	−0.161	(−0.272; −0.051)	−0.035	(−0.171; 0.100)	−0.008	(−0.139; 0.122)
Lag 4	−0.099	(−0.153; −0.046)	−0.157	(−0.240; −0.074)	−0.066	(−0.166; 0.033)	−0.052	(−0.145; 0.041)
Lag 5	−0.100	(−0.228; 0.029)	−0.112	(−0.308; 0.084)	−0.113	(−0.352; 0.125)	−0.109	(−0.339; 0.120)
Overall	−0.306	(−0.564; −0.048)	−0.523	(−0.922; −0.124)	−0.296	(−0.775; 0.184)	−0.070	(−0.522; 0.382)
**PM_2.5_**								
Lag 0	0.321	(−0.070; 0.711)	0.288	(−0.306; 0.882)	0.435	(−0.277; 1.146)	0.164	(−0.538; 0.867)
Lag 1	−0.014	(−0.197; 0.169)	−0.053	(−0.335; 0.229)	0.057	(−0.281; 0.395)	0.008	(−0.314; 0.331)
Lag 2	−0.211	(−0.418; −0.003)	−0.280	(−0.599; 0.039)	−0.156	(−0.541; 0.229)	−0.083	(−0.449; 0.283)
Lag 3	−0.270	(−0.476; −0.064)	−0.393	(−0.708; −0.078)	−0.203	(−0.584; 0.178)	−0.109	(−0.473; 0.255)
Lag 4	−0.191	(−0.363; −0.020)	−0.392	(−0.659; −0.126)	−0.085	(−0.401; 0.232)	−0.070	(−0.370; 0.231)
Lag 5	0.024	(−0.350; 0.399)	−0.278	(−0.855; 0.299)	0.193	(−0.491; 0.876)	0.034	(−0.629; 0.697)
Overall	−0.340	(−1.206; 0.526)	−1.109	(−2.448; 0.231)	0.247	(−1.339; 1.833)	−0.055	(−1.586; 1.476)
**BC**								
Lag 0	0.831	(−0.174; 1.836)	0.968	(−0.592; 2.529)	0.441	(−1.345; 2.227)	0.910	(−0.885; 2.705)
Lag 1	0.339	(−0.103; 0.782)	0.668	(−0.033; 1.370)	0.238	(−0.551; 1.027)	−0.087	(−0.862; 0.688)
Lag 2	−0.085	(−0.710; 0.540)	0.245	(−0.729; 1.219)	−0.733	(−1.842; 0.377)	−0.633	(−1.747; 0.482)
Lag 3	−0.442	(−1.092; 0.208)	−0.302	(−1.311; 0.708)	−0.288	(−1.442; 0.865)	−0.728	(−1.892; 0.436)
Lag 4	−0.731	(−1.218; −0.245)	−0.972	(−1.739; −0.204)	−0.611	(−1.477; 0.256)	−0.373	(−1.234; 0.489)
Lag 5	−0.953	(−1.902; −0.004)	−1.765	(−3.260; −0.271)	−0.973	(−2.657; 0.710)	0.434	(−1.239; 2.106)
Overall	−1.040	(−3.301; 1.220)	−1.157	(−4.795; 2.482)	−1.198	(−5.244; 2.848)	−0.476	(−4.380; 3.428)

Tangará da Serra-MT, Brazil – 2008.

Moreover, for all children, the PDLM 0–5 effect estimates of PM_2.5_ showed reductions for lag 2 (−0.21; 95%CI: −0.42; −0.003), lag 3 (−0.27; 95%CI: −0.48; −0.06), and lag 4 (−0.19; 95%CI: −0.36; −0.02), but there was no overall effect. Additionally, for children aged 6–8, the results revealed reductions for lags 3 and 4.

BC presented associations with PEF. For all children, PDLM 0–5 revealed a reduction only for lag 4 (−0.73; 95%CI: −1.22; −0.24). Furthermore, for children aged 6–8, there were reductions of 0.97 (95%CI: −1.74; −0.20) and 1.77 (95%CI: −3.26; −0.27) in lags 4 and 5, respectively.

### Sensitivity Analysis

Exclusion of outliers hardly affected the results. However, other exposures showed adverse effects, for instance: (i) For single-lag models, PM_10_ lag 3 presented a negative association with PEF for children aged 6–8, and PM_2.5_ lagged by 3 days for all children; (ii) for PDLM, a reduction was observed with PM_2.5_ lag 2 in children aged 6–8.

We found evidence for PM_10_ thresholds. Under the linear parametric spline approach, there were two slopes, one for the effect estimate below the threshold and one for the effect above it. Concerning the former, there were no important findings, except for lags of PM_10_ such as: (i) lag 2: there was a reduction of 0.70 (95%CI: −1.30; −0.99) for exposures below 50 µg/m^3^; (ii) lag 3: the effects of exposures below 60 µg/m^3^, 70 µg/m^3^, and 80 µg/m^3^ were reductions of 0.49 (95%CI: −0.93; −0.05), 0.45 (95%CI: −0.83; −0.07), and 0.40 (95%CI: −0.72; −0.08), respectively; (iii) lag 1–5: reductions for the exposures below 60 µg/m^3^ (−0.63; 95%CI: −1.18; −0.08), 70 µg/m^3^ (−0.55; 95%CI: −0.99; −0.11), and 80 µg/m^3^ (−0.46; 95%CI: −0.84; −0.09).

**Figure 3:**
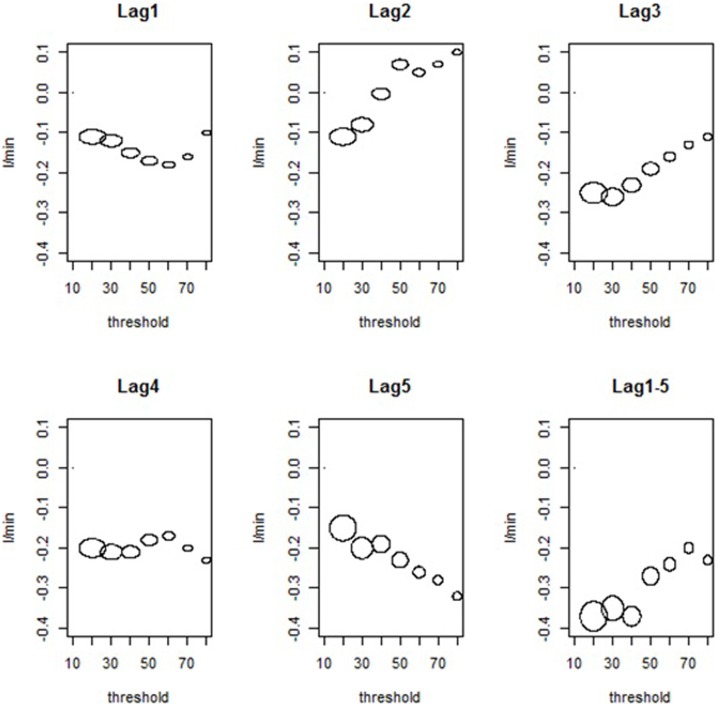
Estimated changes in peak expiratory flow (in l/min) for an increase of 10 µg/m^3^ in PM_10_, above the thresholds for all children. Tangará da Serra-MT, Brazil – 2008.

Figure 3 presents a bubble plot for the estimated effects above the thresholds for all exposures. The size of the bubbles is proportional to the number of days above the thresholds. Negative associations between PEF and PM_10_ were noticed for lags 3, 4, 5, and lag 1–5. More specifically, for lag 3 the associations were important for the following thresholds: 20 µg/m^3^ (−0.25; 95%CI: −0.40; −0.10); 30 µg/m^3^ (−0.26; 95%CI: −0.41; −0.11); and 40 µg/m^3^ (−0.23; 95%CI: −0.40; −0.06). For lag 4, the effects were considerable for the same thresholds, varying from −0.20 µg/m^3^ to −0.21 µg/m^3^ (95%CI: −0.35, −0.08), as well as for 50 µg/m^3^ (−0.18; 95%CI: −0.34; −0.02). Concerning lag 5, there were reductions for all thresholds: (i) 20 µg/m^3^, −0.15 (95%CI: −0.30; −0.01); (ii) 30 µg/m^3^, −0.20 (95%CI: −0.35; −0.04); (iii) 40 µg/m^3^, −0.19 (95%CI: −0.36; −0.03); (iv) 50 µg/m^3^, −0.23 (95%CI: −0.42; −0.05); (v) 60 µg/m^3^, −0.26 (95%CI: −0.47; −0.05); (vi) 70 µg/m^3^, −0.28 (95%CI: −0.53; −0.03); (vii) 80 µg/m^3^, −0.32 (95%CI: −0.62; −0.03). For the cumulative exposure (lag1–5), the reductions were associated with the following thresholds: 20 µg/m^3^ (−0.37; 95%CI: −0.57; −0.16); 30 µg/m^3^ (−0.35; 95%CI: −0.55; −0.14); and 40 µg/m^3^ (−0.37; 95%CI: −0.61; −0.13).

## Discussion

This study's results suggest that air pollution from biomass burning may be a respiratory health risk factor for schoolchildren aged 6 to 15 years old living in the Brazilian Amazon. This association is consistent with the previous panel study undertaken in the municipality of Alta Floresta [10]. The current study extended previous analyses by including PDLM as well as adding the effects of BC and PM_10_ exposures. Further, we explored two methods for analysing panel studies: MEM and univariate time series modelling, which was applied to every child. Although the combined-effect estimates obtained using the univariate approach and those from MEM were similar, their precision was different. However, it was particularly important to show that some students were more vulnerable to air pollution than were others.

Our findings corroborate the results of panel studies describing a linear effect of air pollution on PEF [5], [8], [29]. No association was found with PM_2.5_ or BC in a rural panel study undertaken in the southeast of Brazil to investigate the effects of pre-harvest sugarcane burning on PEF [30]. Systematic reviews of panel studies demonstrated negative pooled effects of PM_10_ and PM_2.5_ on PEF [31], [32], [33].

Although we found associations for all children, the effects were stronger after stratification by age, with PEF decrements for the youngest group, confirming that younger children are more susceptible to air pollution effects [1]. In the Alta Floresta study [10], a negative association of PM_2.5_ with PEF was found for children who studied in the afternoon. However, the authors noted that the majority of children in this group were from 6 to 9 years old. Our results confirm the hypothesis that age is most likely to explain this finding.

A multitude of factors are related to children's PEF. In the present study, PEF measurements were associated with temperature, humidity, BMI, age, sex, and asthma status. Weather conditions can influence children's health [34] but also interfere with pollutants in the atmosphere [35]. Other environmental factors could affect PEF, for instance, time spent outdoors and passive smoking. However, in this study these variables were not important in the modelling, most likely because in the dry season children spend most of their time outdoors when they are not at school because of lack of leisure indoors as well as the high temperatures.

Most adverse effects found in this study were lagged by 3, 4, and 5 days. Lagged effects as well as cumulative effects are expected in this region not only because of the characteristics of Amazon biomass burning (every day during the dry season) but also because of meteorological factors, which allow for pollutants to remain in the atmosphere for long periods of time. The PDLM approach allowed exploring the effect of air pollutants on PEF distributed over time and also the overall effect up to 3 or 5 lagged days. The results revealed negative associations between air pollution and PEF, mainly for PDLM 0–5, which is consistent with the Mexico City panel study that found reductions in children's PEF associated with O_3_, PM_2.5_, and PM_10_ [21]. In the literature, this methodology had been broadly applied in time series studies [26] but not as much in children's panel studies.

The PM_10_ effect was scrutinised using different thresholds. Our results suggest hazardous effects below 50 µg/m^3^. For lag 5, there was a clear negative gradient. The World Health Organisation guidelines for PM_10_ are 20 µg/m^3^ for the annual average and 50 µg/m^3^ for the daily average in urban areas [2]. During the 2008 dry season in Tangará da Serra, approximately 53% of the measurements were above 50 µg/m^3^.

This study could be taken into consideration by the Brazilian Ministry of Health and other health agencies to establish guidelines for health protection in regions where biomass burning takes place. Reducing air pollutant levels is a challenge for local authorities; however, more effective actions should be taken to minimise fires, such as intensified patrolling in the region, heavy fines, policy reforms on taxes and credits, and tougher legislation concerning land occupation conflicts.

Air pollution was measured at the local university campus that is near the study school. In fact, all study children lived within a 5 km distance from the university campus. We believe that the device location did not sub or super estimated the observed adverse effects because particles emitted through biomass burning have relatively long resident time in the atmosphere and can be transported over long distances, crossing international boundaries [18]. This study would have benefited from a more specific questionnaire, for instance to evaluate patterns of physical activity and time spent outdoors.

In conclusion, this study showed a negative association between exposure to air pollution and PEF in schoolchildren living in Tangará da Serra. The analysis per child indicated that age was an effect modifier and that air pollution mostly affects younger children.
